# Livestock-Associated Methicillin-Resistant *Staphylococcus aureus* (LA-MRSA) Isolates of Swine Origin Form Robust Biofilms

**DOI:** 10.1371/journal.pone.0073376

**Published:** 2013-08-09

**Authors:** Tracy L. Nicholson, Sarah M. Shore, Tara C. Smith, Timothy S. Fraena

**Affiliations:** 1 National Animal Disease Center, Agricultural Research Service, United States Department of Agriculture, Ames, Iowa, United States of America; 2 Center for Emerging Infectious Diseases, Department of Epidemiology, College of Public Health, University of Iowa, Iowa City, Iowa, United States of America; 3 Department of Veterinary Diagnostic and Production Animal Medicine, College of Veterinary Medicine, Iowa State University, Ames, Iowa, United States of America; National Institutes of Health, United States of America

## Abstract

Methicillin-resistant *Staphylococcus aureus* (MRSA) colonization of livestock animals is common and prevalence rates for pigs have been reported to be as high as 49%. Mechanisms contributing to the persistent carriage and high prevalence rates of livestock-associated methicillin-resistant *Staphylococcus aureus* (LA-MRSA) strains in swine herds and production facilities have not been investigated. One explanation for the high prevalence of MRSA in swine herds is the ability of these organisms to exist as biofilms. In this report, the ability of swine LA-MRSA strains, including ST398, ST9, and ST5, to form biofilms was quantified and compared to several swine and human isolates. The contribution of known biofilm matrix components, polysaccharides, proteins and extracellular DNA (eDNA), was tested in all strains as well. All MRSA swine isolates formed robust biofilms similar to human clinical isolates. The addition of Dispersin B had no inhibitory effect on swine MRSA isolates when added at the initiation of biofilm growth or after pre-established mature biofilms formed. In contrast, the addition of proteinase K inhibited biofilm formation in all strains when added at the initiation of biofilm growth and was able to disperse pre-established mature biofilms. Of the LA-MRSA strains tested, we found ST398 strains to be the most sensitive to both inhibition of biofilm formation and dispersal of pre-formed biofilms by DNaseI. Collectively, these findings provide a critical first step in designing strategies to control or eliminate MRSA in swine herds.

## Introduction

*Staphylococcus aureus* is an opportunistic pathogen that can cause serious disease in humans, ranging from skin and soft tissue infections to invasive infections of the bloodstream, heart, lungs and other organs [1]. It is frequently carried asymptomatically on the skin and in the anterior nares. A 2003-2004 survey found that approximately 30% of the U.S. population was colonized by *S. aureus* and approximately 1.5% of the U.S. population was found to carry methicillin-resistant *S. aureus* (MRSA) [2]. First identified in 1961, MRSA is a major cause of healthcare-related infections, responsible for a significant proportion of nosocomial infections worldwide [3–5]. Recently, deaths from MRSA infections in the U.S. have eclipsed those from many other infectious diseases, including HIV/AIDS [6]. In the mid-1990s, new strains of MRSA emerged, causing infections in healthy individuals who had no recent contact with healthcare facilities [7]. These community-associated MRSA (CA-MRSA) strains are genetically distinct from the hospital-associated MRSA (HA-MRSA) strains and are typically more virulent, owing to the presence of a variety of toxins, such as Pantón-Valentine leukocidin (PVL) [1,5,8]. CA-MRSA has now spread worldwide and is beginning to replace HA-MRSA strains in healthcare facilities [5,9].

*S. aureus* can also infect a variety of animal species and is one of the many pathogens known to cause mastitis in cattle [10]. Not surprisingly, MRSA has also been found among animal populations and was first isolated in 1972 from Belgian cows with mastitis [11]. Frequently, the MRSA strains isolated from animals resemble human strains and presumably were transferred from their human caretakers [10,11]. Recently however, a new lineage has been found in livestock. First identified in pigs in The Netherlands in 2003 [12,13], these livestock-associated MRSA (LA-MRSA) isolates are genetically distinct from human isolates [14]. Most LA-MRSA from swine can be assigned by multilocus sequence typing (MLST) to a single sequence type, ST398 [15]. Since its discovery, ST398 MRSA has been shown to be widespread, detected on pig farms in The Netherlands, Germany, Belgium, Denmark, Portugal, Canada and the United States [13,16–28]. In the United States, Smith and colleagues reported forty-nine percent of the animals and 45% of the workers examined on farms in Iowa and Illinois were found to carry MRSA and all isolates typed from both swine and workers were found to be ST398 [16]. ST398 MRSA can be transmitted from pigs to humans as numerous studies have shown that farm workers and others working in close contact with pigs are at significant risk for colonization by ST398 [14,16,28–34]. Human carriage of ST398 is typically asymptomatic, however sporadic cases of serious disease have been reported [15,35–38]. ST398 MRSA has also been found in retail meat products in Europe, Canada and the United States [26,39–42], although it is unclear whether this poses a significant risk for transmission to the general public [14].

Recently, key phenotypic and genomic distinguishing features have been identified in human MRSA and LA-MRSA isolates. For example, transfer of LA-MRSA isolates beyond the immediate animal-exposed human contacts has rarely been observed and persistent nasal colonization is infrequently detected in individuals without direct animal exposure [31]. Consistent with this, LA-ST398 MRSA isolates have been reported to be less transmissible among humans than HA-MRSA isolates [43]. Using *in vitro* binding assays, ST398 MRSA isolates were reported to bind significantly less to human skin keratinocytes and keratin compared to human MSSA isolates [44]. Genome level distinguishing features identified in human MRSA and LA-MRSA isolates include mobile genetic elements (MGEs), such as the immune evasion cluster (IEC) of genes carried on the ϕ13 family of bacteriophage, which are found in nearly all human isolates, but rarely found in LA-MRSA isolates [44–46]

LA-MRSA strains, largely comprising MLST type ST398, currently represent the largest reservoir of MRSA outside of a hospital setting [47]. Therefore, strategies to eliminate or decrease the prevalence of these strains in swine herds are a public health priority. Outside of the previously mentioned *in vitro* binding assays [44], no other phenotypic studies have been undertaken to specifically investigate virulence or survival mechanisms associated with LA-MRSA strains. It is well established that biofilm formation is an important contributing factor in chronic human infections caused by *S. aureus*. Biofilms are adherent communities of bacteria encased within a complex matrix that protects the encased bacterial community from a variety of environmental stresses such as shear flow forces, antimicrobial compounds, and host immune and clearance mechanisms [48,49]. We hypothesized that if biofilms are an important survival trait for this species, then this phenotype will be conserved in LA-MRSA strains. In this study, we examined a collection of methicillin-sensitive *S. aureus* (MSSA) and MRSA isolates of different sequence types (STs) from swine and retail meat sources for their ability to form biofilms. Given the genotypic and phenotypic differences observed between human MRSA and LA-MRSA isolates, we then compared the biofilms formed by the LA-MSSA and LA-MRSA strains to biofilms formed by laboratory MSSA and MRSA strains and human MRSA isolates, including HA-MRSA (USA100) and CA-MRSA (USA300) strains. To gain further insights into the mechanisms responsible for biofilm development, we additionally tested the contribution of known biofilm matrix components, polysaccharides, proteins and extracellular DNA (eDNA), in these LA-MRSA strains. 

## Materials and Methods

### Bacterial Strains and Growth Conditions

The bacterial strains used in this study are listed in Table 1. *S. aureus* strains were grown in tryptic soy broth (Becton-Dickinson, Sparks, MD) supplemented with 0.5% glucose and 3% NaCl (TSB-GN). *Staphylococcus epidermidis* strains were grown in tryptic soy broth supplemented with 0.5% glucose (TSB-G). All strains were grown at 37° C and maintained on tryptic soy agar plates (Becton-Dickinson, Sparks, MD).

**Table 1:** Bacterial strains used in this study.

**Strain**	**Isolated From:**	**Methicillin Sensitivity**	**MLST**	***spa* Type**	**Source or Reference**
*S. aureus*
Newman	human	MSSA			ATCC
ATCC 29213	human	MSSA			ATCC
SH1000		MSSA			[107]
MN06	pig	MSSA	ST5	t002	[16]
MN55	pig	MSSA	ST9	t337	[16]
MN56	pig	MSSA	ST9	t337	[16]
ATCC 43300	human	MRSA			ATCC
USA100	human	MRSA	ST5	t002	T. Smith
USA300	human	MRSA	ST8	t008	T. Smith
TCH1516 (USA300)	human	MRSA	ST8	t008	[86]
HU01010T	human	MRSA	ST398	t034	[16]
HU01011N	human	MRSA	ST398	t034	[16]
MRS910	pig	MRSA	ST398	t034	T. Fraena
MRS913	swine facility	MRSA	ST398	t034	T. Fraena
MRS922	pig	MRSA	ST398	t034	T. Fraena
MRS926	pig	MRSA	ST398	t034	T. Fraena
MRS927	swine facility	MRSA	ST398	t034	T. Fraena
MRS879	pig	MRSA	ST5	t002	T. Fraena
MRS935	pig	MRSA	ST5	t002	T. Fraena
MRS1008	pig	MRSA	ST5	t548	T. Fraena
IA63	pork sample	MRSA	ST398	t034	[42]
IA91	pork sample	MRSA	ST398	t034	[42]
IA97	pork sample	MRSA	ST398	t034	[42]
MN48	pork sample	MRSA	ST398	t034	[42]
MN135	pork sample	MRSA	ST398	t034	[42]
NJ101	pork sample	MRSA	ST398	t034	[42]
P2(HPH1)	pork sample	MRSA	ST398	t034	[108]
T2(T2TGT)	turkey meat	MRSA	ST398	t034	[108]
T3(TGT)	turkey meat	MRSA	ST398	t034	[108]
*S. epidermidis*	
1457	human				[109]
NJ9709	human				[110]

### Microtiter Plate Assay for Biofilm Formation

Biofilm formation was assessed using a microtiter plate assay as previously described [50,51], except the surface of the microtiter plates used were coated with porcine plasma to increase biofilm adherence to the plate ( [50,51]; data not shown). Briefly, Costar 3596 plates (Corning Life Sciences, Lowell, MA) were coated with lyophilized porcine plasma (Sigma, St. Louis, MO) by incubating each well with 100 µl of a 20% porcine plasma solution in 0.05M carbonate-bicarbonate buffer (pH 9.6) overnight at 4° C. The plasma solution was removed from the plate immediately prior to use. Overnight cultures of all strains were diluted to an OD_600_ of 0.05 in fresh media and 100 µl was added to each well. For each experimental plate, 3 wells were used for each strain representing one biological replicate. The plates were incubated statically for 24 hours in a humidified 37° C incubator. The cultures were aspirated from the plate wells and each well was washed 3 times with 200 µl sterile PBS. The biofilms were fixed by the addition of 150 µl 100% ethanol and dried for 10 minutes. Biofilms were stained by the addition of 150 µl 0.1% crystal violet (CV) (Sigma, St. Louis, MO) to each well and incubated for 15 minutes. CV was removed and the wells were washed 3 times with 200 µl sterile PBS and the plate allowed to dry overnight. Bound CV dye was eluted by incubation for 10 minutes with 150 µl 100% ethanol. 120 µl of the elution was transferred to a new 96-well plate and biofilm biomass was quantified by measuring the absorbance at 538 nm (A_538_) in a microplate reader (SpectraMax M5, Molecular Devices, Sunnyvale, CA). At least 3 independent biological replicates were performed and the overall average A_538_ was determined from all biological replicates.

### Inhibition of Biofilm Formation

For the inhibition studies, the microtiter plate assay was performed as described above except that the treated bacterial cells were diluted in media containing one of the following: 100 µg/ml Proteinase K (Roche Applied Science, Indianapolis, IN), 140 U/ml DNaseI (Roche Applied Science, Indianapolis, IN) or 40 µg/ml Dispersin B (DspB) [52] (Kane Biotech, Winnipeg, Canada). Control bacterial cells were diluted in media alone. Prior to first wash, absorbance at 600 nm was measured using a microplate reader to ensure that addition of the enzyme did not inhibit cell growth or viability (data not shown).

### Dispersal of Pre-Formed Biofilms

Biofilms were grown in microtiter plates as described above. Following growth for 24 hours at 37° C, the biofilms were rinsed once with 200 µl sterile PBS and incubated for 2 hours at 37° C with 100 µl of one of the following: Proteinase K (100 µg/ml in 10 mM Tris-HCl, pH 7.5), DNaseI (140 U/ml in culture medium) or DspB (40 µg/ml in PBS). Control wells were treated with 100 µl of the appropriate buffer. Following enzymatic treatment, the wells were washed and stained as described above.

### Statistical Analysis

For quantification of biofilm formation using the microtiter plate assay, a two-way mixed model ANOVA with a *post-hoc* comparison of the means of each strain evaluated using the Bonferroni method was used to determine significance. For the inhibition and dispersal assays, the 2-tailed Student’s *t* test was used to determine the significance of the difference between the means of the treated versus untreated groups. A *p*-value less than 0.05 was considered significant.

### RNA Isolation

For RNA isolation, biofilm cultures of *S. aureus* strains were grown in BD Falcon 6-well plates (BD Labware, Franklin Lakes, NJ). The plates were pre-coated with a 20% porcine plasma solution (2.5 ml per well) by overnight incubation at 4° C as described for the microtiter plate assay. Overnight cultures of all strains were diluted to an OD_600_ of 0.05 in fresh TSB-GN and 2.5 ml was added to each well. For each experimental sample (biological replicate), 3 wells were used for each strain. For each strain, 2 samples were prepared. The plates were incubated statically for 24 hours at 37° C in a humidified incubator. The culture media was removed by aspiration and each well was washed 3 times with 3 ml sterile PBS to remove unattached bacteria.

As obtaining RNA from biofilm samples can be difficult, a customized RNA extraction protocol based on chemical and mechanical lysis, organic extraction and silica membrane purification was developed and optimized for these samples, drawing from methods developed for RNA isolation from *S. epidermidis* biofilms [53,54]. After washing the biofilm cultures, 3 ml TRI Reagent Solution (Ambion, Carlsbad, CA) was added to each well of the 6-well plate and incubated for 15 minutes at room temperature. A cell scraper was used to ensure complete detachment of the biofilm from the plate surface and the mixture transferred to a 15 ml Falcon tube (BD Labware, Franklin Lakes, NJ) and mixed thoroughly. For each strain, biofilms in TRI Reagent from 3 wells were combined into one sample for subsequent RNA purification steps; 2 samples were obtained for each strain. At this point, samples were stored at -80° C prior to further processing. Next, 1 ml of the bacteria in TRI Reagent was added to a 2 ml screw-cap tube containing 0.5 g of acid-washed 0.25 mm carbide beads (MO BIO Laboratories, Carlsbad, CA) and heated to 60° C for 20 minutes with periodic mixing. This was followed by vortexing the samples for 20 minutes at maximum speed. The carbide beads were pelleted by centrifugation (10,000 x *g*, 1 minute) and the TRI Reagent lysate transferred to a 1.5 ml tube. Phase separation was performed by addition of 0.2 volumes chloroform and centrifugation at 12,000 x *g* for 15 minutes at 4° C. The aqueous phase was transferred to a new 1.5 ml tube and mixed with an equal volume of 95% ethanol. The Direct-zol RNA MiniPrep Kit (Zymo Research, Irvine, CA) was used according to the manufacturer’s instructions and total RNA was eluted in 25-50 µl RNase-free water. Concentration and purity of the total RNA was assessed by spectrophotometry using a NanoDrop 1000 (Thermo, Fisher Scientific, Wilmington, DE). Total RNA samples that were too dilute at this point were concentrated using the RNA Clean & Concentrator-5 kit (Zymo Research, Irvine, CA) according to the manufacturer’s instructions and total RNA was eluted in 10 µl RNase-free water.

### cDNA Synthesis and Quantitative Real-Time PCR

Primers for quantitative Real-Time PCR (qPCR) detection of 16S rRNA, *icaA, icaR, nuc1* and *nuc 2* were designed using Primer Express 2.0 software (Applied Biosystems, Foster City, CA) and are listed in Table 2. Coding sequences for the target genes were obtained from the genome sequences of the *S. aureus* strains Newman (GenBank accession number NC_009641.1), NCTC 8325 (GenBank accession number NC_007795.1), TCH1516 (GenBank accession number NC_010079.1), USA300 FPR3757 (GenBank accession number NC_007793.1), JKD6008 (GenBank accession number NC_017341.1), ST398 SO385 (GenBank accession number AM990992.1), 08BA02176 (GenBank accession number CP003808.1), and LGA251 (GenBank accession number NC_017349.1), and aligned using Geneious 5.0.2 (Biomatters, available from http://www.geneious.com/) to generate consensus sequences for primer design. Primer efficiencies were tested using dilutions of purified genomic DNA and determined to be similar for all *S. aureus* strains listed in Table 1.

**Table 2:** qPCR Primers used in this study.

**Target**	**Primer Name**	**Sequence**
16S rRNA	16S-SARTfor	GAGGGTGATCGGCCACACT
	16S-SARTrev	ACTGCTGCCTCCCGTAGGA
*icaA*	icaA-SARTfor	AATTGGCTGTATTAAGCGAAGTCA
	icaA-SARTrev	GAGTGAAGACACCCGAAATAGTATTG
*icaR*	icaR-SARTfor	GAAAGTTGGTATTTGAAAGCATCCTT
	icaR-SARTrev	ATTTAGTAGCGAATACACTTCATCTTTGA
*nuc1*	nuc1-SARTfor	GCTTAGCGTATATTTATGCTGATGGA
	nuc1-SARTrev	TTTAGCCAAGCCTTGACGAACT
*nuc2*	nuc2-SARTfor	GTATGTACAATAAGGAATTAGTGGAAAAGG
	nuc2-SARTrev	CTGTTGTTTAGCTTTATTTTGTGCTTCT

Total RNA samples were treated with DNaseI to remove any residual genomic DNA. Briefly, 300 ng RNA was incubated with 1 µl Amplification Grade DNaseI (Invitrogen, Carlsbad, CA) in a total volume of 10 µl for 15 minutes at 25° C. The DNaseI enzyme was inactivated by addition of 1 µl 25 mM EDTA and incubation at 65° C for 10 minutes. The DNase-treated RNA sample was reverse transcribed using 100 ng random primers and SuperScriptIII reverse transcriptase (Invitrogen, Carlsbad, CA) according to the manufacturer’s instructions. The resulting cDNA was diluted 1:250 (for 16S rRNA qPCR) or 1:50 (for other targets) in water and 5 µl of these dilutions was used for qPCR in reactions containing 400 nM primers and SYBR Green PCR Master Mix (Applied Biosystems, Foster City, CA) in a 25 µl reaction volume. qPCR runs were performed on an Applied Biosystems 7300 Real Time PCR System (Applied Biosystems, Foster City, CA). Reactions lacking reverse transcriptase enzyme were performed to verify the absence of genomic DNA contamination and dissociation curve analysis of qPCR products was performed to confirm the lack of non-specific products. Relative expression of the *icaA, icaR, nuc1* and *nuc2* mRNA was determined using the 2^-ΔCt^ method [55] using the 16S rRNA as the endogenous control. Analysis was performed using the Sequence Detection Software version 1.3.1 with RQ Study Application (Applied Biosystems, Foster City, CA). Statistical analysis was performed on the data from each primer set using a one-way ANOVA with a *post-hoc* comparison of the means of each strain using the Tukey-Kramer test using GraphPad Prism 5.04 for Windows (GraphPad Software, San Diego, CA). A P value less than 0.05 was considered significant.

### Production of Extracellular Proteases

Protease activity in conditioned culture media was assessed using a fluorescence-based assay for protease activity. Overnight cultures of all strains were diluted to an OD_600_ of 0.05 in TSB-GN (or TSB-G for *S. epidermidis* strains). Biofilm cultures were grown in 6-well plates as described above for 24 hours and the conditioned media recovered. Planktonic cultures were started at an OD_600_ of 0.05 and grown for 22 hours at 37° C in a shaking incubator. Bacterial cells were pelleted by centrifugation and 2.5 ml of the conditioned media recovered. Conditioned media from biofilm and planktonic cultures was filtered with 0.22 µm syringe filters and concentrated using Amicon Ultra-0.5 3K centrifugal filter units (EMD Millipore, Billerica, MA). 10 µl of the concentrated media was tested using the SensoLyte Red Protease Assay Kit (AnaSpec, Inc., Fremont, CA) according to the manufacturer’s instructions with a 2.5 hour incubation at 37° C. Fluorescence intensity (excitation 546 nm/emission 575 nm) was measured in a SpectraMax M5 microplate reader (Molecular Devices, Sunnyvale, CA) and is proportional to protease activity in the sample. Sterile TSB-GN medium concentrated in the same manner as the conditioned media samples was used as a control.

### Production of Extracellular Nucleases

Conditioned media from biofilm and planktonic cultures was obtained as described above. BBL DNase Test Agar with Methyl Green plates (BD, Sparks, MD) were used to detect nuclease activity in the conditioned media samples. Small (approximately 2 mm diameter) wells were cut into the agar into which 10 µl of the conditioned media was deposited. The plates were incubated overnight at 37° C. A clear zone in the green agar indicated the presence of nuclease activity in the conditioned media samples [56,57]. 

## Results

### MRSA isolates from swine form robust biofilms

**Figure 1:**
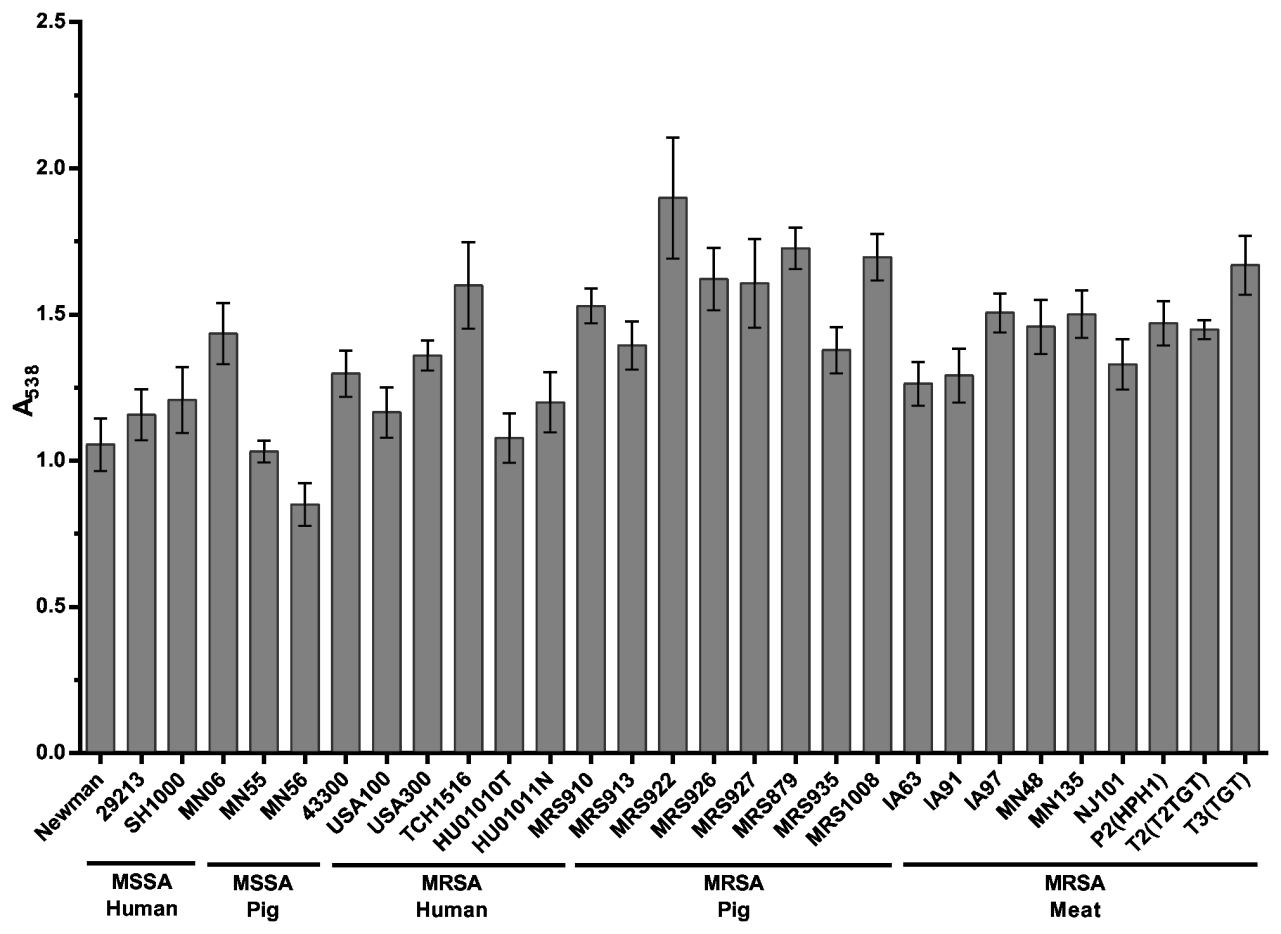
Biofilm forming capacity of *S. aureus* isolates. Strains tested are shown along the x-axis and grouped based on methicillin-sensitivity and isolation source. The indicated strains were grown statically for 24 hours. Biofilm formation was quantified by standard microtiter assays and measuring the absorbance at 538 nm, plotted along the y-axis. Bars represent the average absorbance obtained from at least 3 independent plates representing biological replicates. Error bars represent +/- the SEM.

To experimentally address whether swine LA-MRSA strains form robust biofilms similar to human MRSA strains, biofilm formation was quantified by standard microtiter crystal violet assays [50,51] in a collection of methicillin-sensitive *S. aureus* (MSSA) and MRSA isolates of different sequence types (STs) from swine and retail meat sources. Previous reports have shown that consistent biofilm formation by human clinical strains can be obtained by using tryptic soy broth medium supplemented with 0.5% glucose and 3% NaCl (TSB-GN) and using polystyrene microtiter plates pre-coated with 20% human plasma [50,57]. As a preliminary test, a subset of strains representing human and porcine origin MSSA and MRSA strains were selected and tested for biofilm formation under these conditions and biofilm formation on plates pre-coated with either 20% human or 20% porcine plasma was compared (Figure S1). The use of human and porcine plasma produced similar effects on biofilm formation by the strains tested, and the effect did not appear to depend upon host origin. Since biofilm formation by a range of strains was supported by growth in TSB-GN on 20% porcine plasma-coated microtiter plates, these conditions were chosen for all subsequent assays. As shown in Figure 1, no statistically significant differences were observed in the capacity to form biofilms in all LA-MRSA strains tested compared to the human MSSA and MRSA strains. Further, no statistically significant differences were observed among any isolates and MLST types tested. These data demonstrate that swine LA-MRSA strains form robust biofilms similar to human MRSA strains, including clinical HA-MRSA (USA100) and CA-MRSA (USA300) strains.

### Inhibition of biofilm formation by enzymatic treatment

Recent studies suggest that for *S. aureus* biofilms, the extracellular matrix consists of proteins, DNA, and/or polysaccharide (poly-β(1,6)-N-acetyl-D-glucosamine or PNAG, also referred to as the polysaccharide intercellular adhesin or PIA) [58–61]. The relative importance of these components in the matrix structure routinely varies between bacterial species and between strains within the same species [58,59,61]. Enzymes capable of breaking down the different matrix components can inhibit or prevent biofilm formation. To begin addressing the importance and functional role of these components in swine LA-MRSA strains, we tested the ability of Proteinase K, DNaseI and dispersion B (DspB) to inhibit biofilm formation.

**Figure 2:**
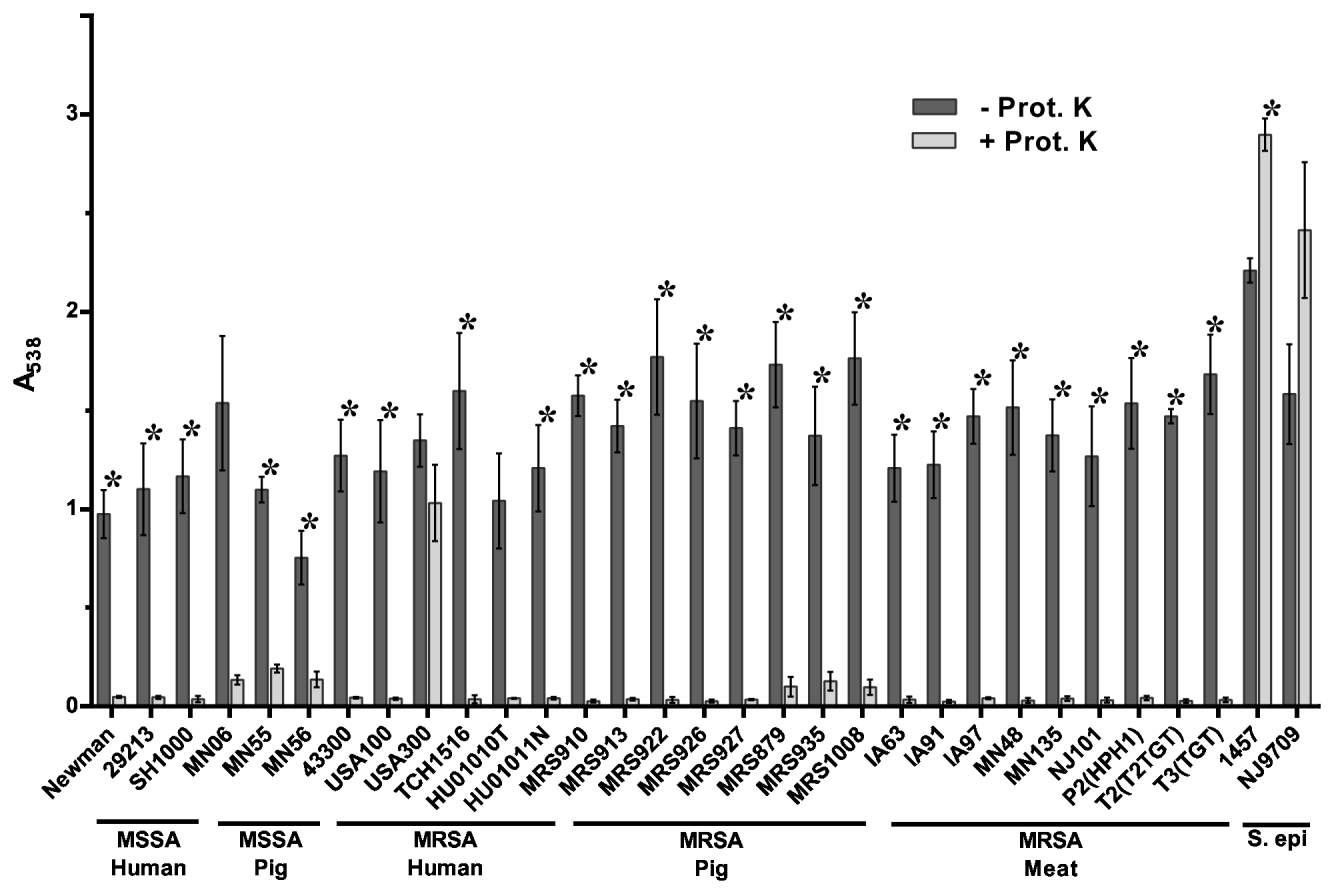
Inhibition of biofilm formation by Proteinase K. Strains tested are shown along the x-axis and grouped based on methicillin-sensitivity and isolation source. The indicated strains were grown statically for 24 hours in media alone (- Prot. K) or in media supplemented with 100 µg/ml Proteinase K (+ Prot. K). Biofilm formation was quantified by standard microtiter assays and measuring the absorbance at 538 nm, plotted along the y-axis. Bars represent the average absorbance obtained from at least 3 independent plates representing biological replicates; error bars represent the SEM. Asterisks (*) denote a *p*-value less than 0.05 between the treated and untreated groups.

When Proteinase K was added to the media at the time of inoculation, biofilm formation was significantly inhibited in the majority of strains tested, including swine LA-MRSA strains (Figure 2). This indicates that proteinaceous material forms a significant component of the biofilm matrix in these strains. The addition of Proteinase K did not significantly inhibit biofilm formation by strains MN06, HU01010T, and USA300. The lack of significant inhibition observed by strains MN06 and HU01010T is due to the large standard deviation among the biological replicates for these strains. Despite the lack of significance, the addition of Proteinase K resulted in a 91% reduction in biofilm formation by strain MN06, and 96% reduction in biofilm formation by HU01010T, while only resulting in a 23% reduction for strain USA300. Interestingly, the two CA-MRSA (USA300) strains tested here differed in their sensitivity to Proteinase K. We found *S. aureus* strain USA300 to be resistant to the effect of Proteinase K under these conditions, whereas strain TCH1516, which is also considered a USA300-type strain, was found to be sensitive to Proteinase K (Figure 2). In contrast to the *S. aureus* strains, biofilm formation by the *S. epidermidis* strains 1457 and NJ9709 was not sensitive to Proteinase K inhibition (Figure 2). In fact, addition of Proteinase K seemed to cause a significant increase in biofilm formation by *S. epidermidis* strain 1457 (Figure 2).

**Figure 3:**
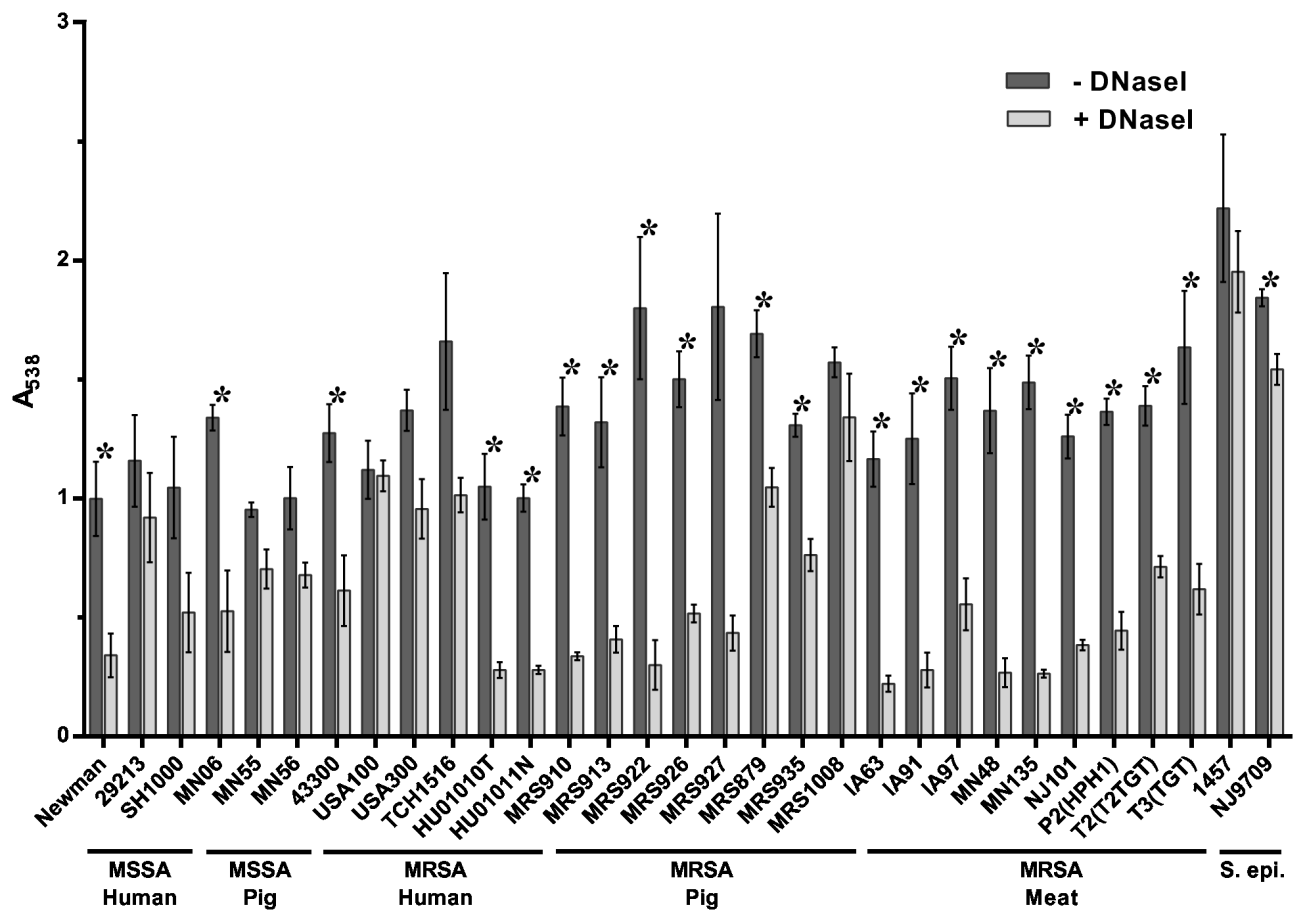
Inhibition of biofilm formation by DNaseI. Strains tested are shown along the x-axis and grouped based on methicillin-sensitivity and isolation source. The indicated strains were grown statically for 24 hours in media alone (- DNaseI) or in media supplemented with 140 U/ml DNaseI (+ DNaseI). Biofilm formation was quantified by standard microtiter assays and measuring the absorbance at 538 nm, plotted along the y-axis. Bars represent the average absorbance obtained from at least 3 independent plates representing biological replicates; error bars represent the SEM. Asterisks (*) denote a *p*-value less than 0.05 between the treated and untreated groups.

Treatment by DNaseI has been shown to inhibit biofilm formation by a number of bacterial species, including *S. aureus* [59,61–63]. As shown in Figure 3, addition of DNaseI to the culture medium at the time of inoculation had a varying effect on biofilm formation, with the tested strains displaying a range of sensitivity. This effect ranged from little to no effect in USA100, with 2% reduction in biofilm formation, to strong, nearly complete inhibition of biofilm formation in LA-MRSA strain MRS922 with 83% reduction (Figure 3 and Table 3). As shown in Table 3, the ST398 strains were the most strongly inhibited by DNaseI (displaying approximately a 50% or greater reduction in biofilm biomass), whereas the strains from other MLST types had a much smaller reduction in biofilm biomass. Together, the data indicate that while extracellular DNA (eDNA) is a component of the biofilm matrix, the role of this component may be more significant in some MLST types of *S. aureus* than in others. Similar to the *S. aureus* strains tested, biofilm formation by the *S. epidermidis* strains 1457 and NJ9709 differed in their sensitivity to DNaseI treatment, with DNaseI significantly inhibiting biofilm formation in *S. epidermidis* NJ9709 and having little to no effect in *S. epidermidis* 1457.

**Table 3:** Biofilm biomass reduction by DNaseI.

**Inhibition**				**Dispersal**		
**Strain**	**ST**	**% Reduction**		**Strain**	**ST**	**% Reduction**
USA100	5	2%		USA100	5	9%
MRS1008	5	15%		USA300	8	21%
MN55	9	26%		MRS1008	5	36%
USA300	8	30%		TCH1516	8	37%
MN56	9	32%		MRS935	5	38%
MRS879	5	38%		MN56	9	47%
TCH1516	8	39%		MN55	9	49%
MRS935	5	42%		MRS879	5	53%
T2(T2TGT)	398	49%		T2(T2TGT)	398	62%
MN06	5	61%		IA97	398	77%
T3(TGT)	398	62%		MN06	5	78%
IA97	398	63%		MRS913	398	85%
MRS926	398	66%		T3(TGT)	398	87%
P2(HPH1)	398	67%		MRS910	398	90%
MRS913	398	69%		P2(HPH1)	398	90%
NJ101	398	70%		HU01011N	398	91%
HU01011N	398	72%		HU01010T	398	91%
HU01010T	398	73%		MN48	398	93%
MRS910	398	76%		MRS926	398	93%
MRS927	398	76%		MRS927	398	93%
IA91	398	78%		IA91	398	93%
MN48	398	80%		NJ101	398	94%
IA63	398	81%		IA63	398	94%
MN135	398	82%		MRS922	398	94%
MRS922	398	83%		MN135	398	95%

**Figure 4:**
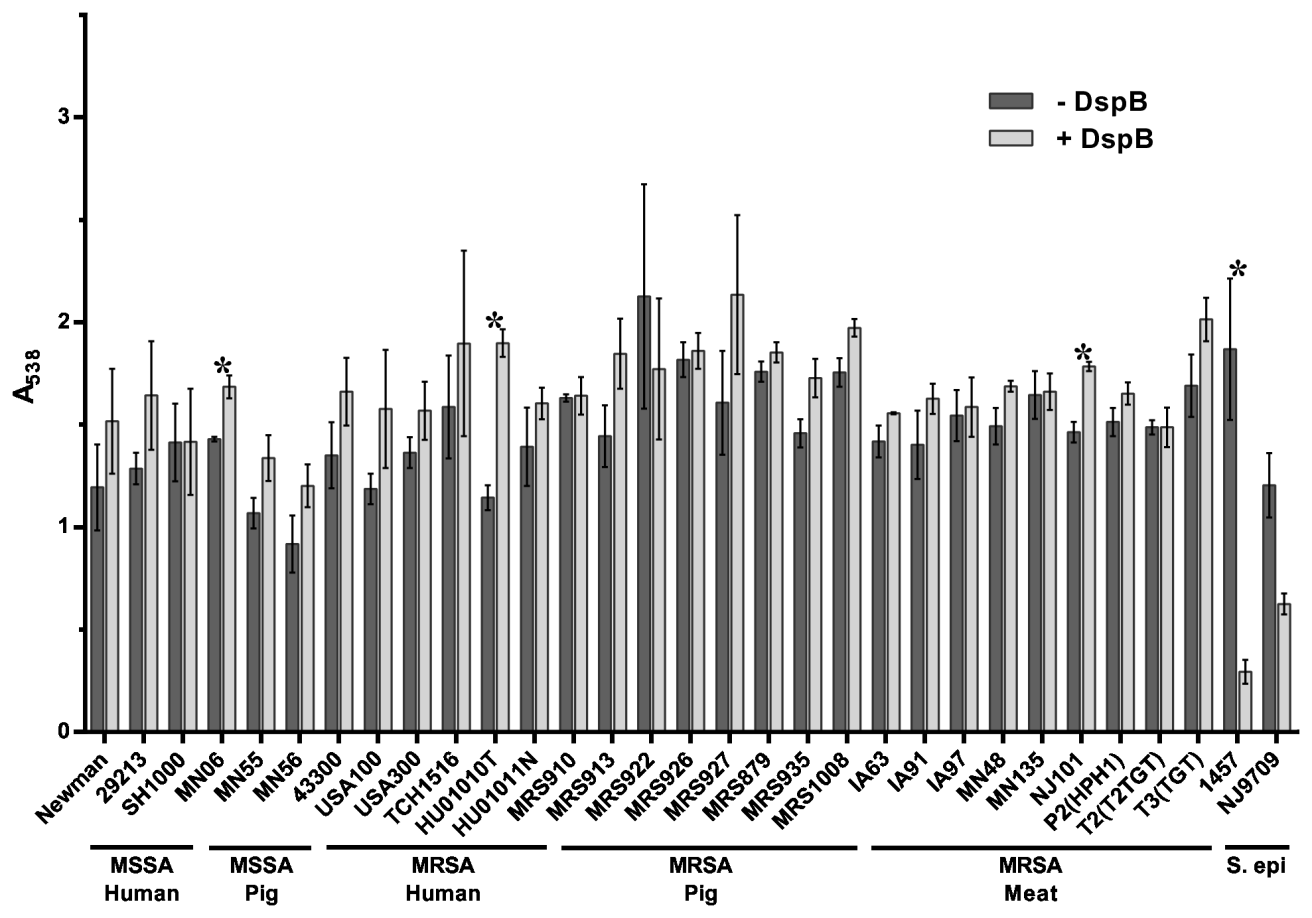
Inhibition of biofilm formation by DspB. *S. aureus* strains tested are shown along the x-axis and grouped based on methicillin-sensitivity and isolation source. *S. epidermidis* (S. epi) strains tested are shown along the x-axis and grouped together. The indicated strains were grown statically for 24 hours in media alone (- DspB) or in media supplemented with 40 µg/ml DspB (+ DspB). Biofilm formation was quantified by standard microtiter assays and measuring the absorbance at 538 nm, plotted along the y-axis. Bars represent the average absorbance obtained from at least 3 independent plates representing biological replicates; error bars represent the SEM. Asterisks (*) denote a *p*-value less than 0.05 between the treated and untreated groups.

Dispersion B (DspB) is an enzyme first isolated from *Actinobacillus actinomycetemcomitans* that breaks down the polysaccharide PNAG or PIA [52] and has been shown to inhibit biofilm formation in a number of bacterial species [64]. The polysaccharide PNAG or PIA is synthesized by the products of the *S. aureus* intercellular adhesion (ica) locus [65]. Given that *S. aureus* is known to form biofilms through both *ica*-dependent and *ica*-independent mechanisms [66,67], we sought to determine the functional role of PIA in the biofilm matrix of swine LA-MRSA strains by testing whether or not the PNAG-degrading enzyme DspB could inhibit biofilm formation in these strains. When added to the culture medium at the time of inoculation, DspB did not inhibit biofilm formation by any of the *S. aureus* strains tested (Figure 4). In contrast, biofilm formation by the *S. epidermidis* strains 1457 and NJ9709 was strongly inhibited by DspB. This is consistent with previous findings and demonstrates that the polysaccharide PNAG does not play as significant a structural role in *S. aureus* as it does in *S. epidermidis* [59].

### Dispersal of pre-formed biofilms by enzymatic treatment

**Figure 5:**
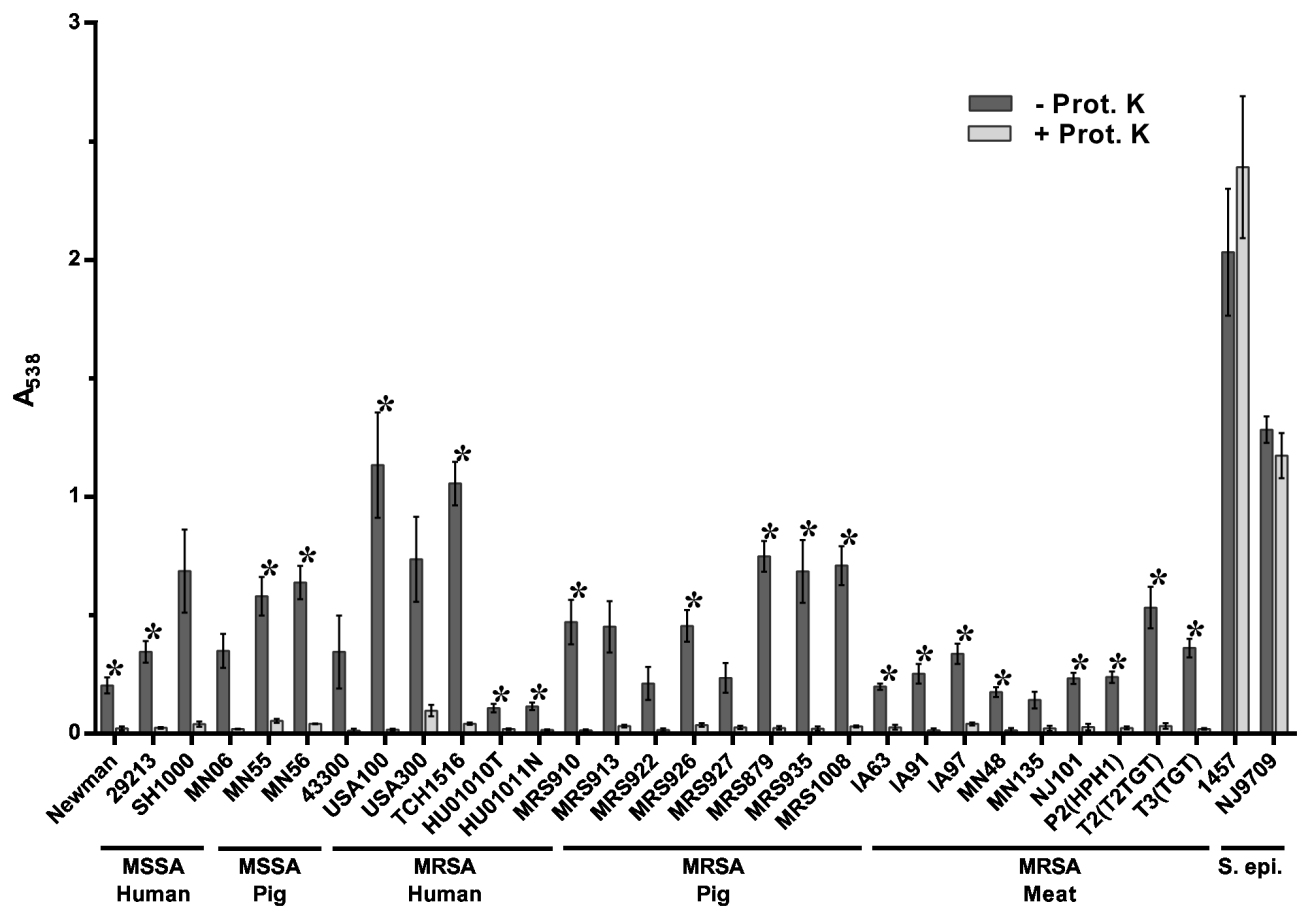
Dispersal of established biofilms by Proteinase K. Strains tested are shown along the x-axis and grouped based on methicillin-sensitivity and isolation source. The indicated strains were grown statically for 24 hours to allow biofilm formation. Wells were washed and treated with buffer alone (- Prot. K) or 100 µg/ml Proteinase K (+ Prot. K) for 2 hours. Biofilm formation was then quantified by standard microtiter assays and measuring the absorbance at 538 nm, plotted along the y-axis. Bars represent the average absorbance obtained from at least 3 independent plates representing biological replicates; error bars represent the SEM. Asterisks (*) denote a *p*-value less than 0.05 between the treated and untreated groups.

To further characterize the role of proteins, DNA, and/or polysaccharide in biofilms, specifically the contribution of each to the development of late stage biofilms, we tested the ability of Proteinase K, DNaseI and DspB to disrupt statically established mature biofilms. Figure 5 shows 24 hour biofilms formed in microtitre plates after a 2 hour treatment with Proteinase K. Incubation of these pre-formed and mature biofilms with Proteinase K caused significant detachment in nearly all *S. aureus* strains tested (Figure 5). In contrast, Proteinase K caused little to no detachment in mature biofilms of *S. epidermidis* strains 1457 and NJ9709 (Figure 5).

**Figure 6:**
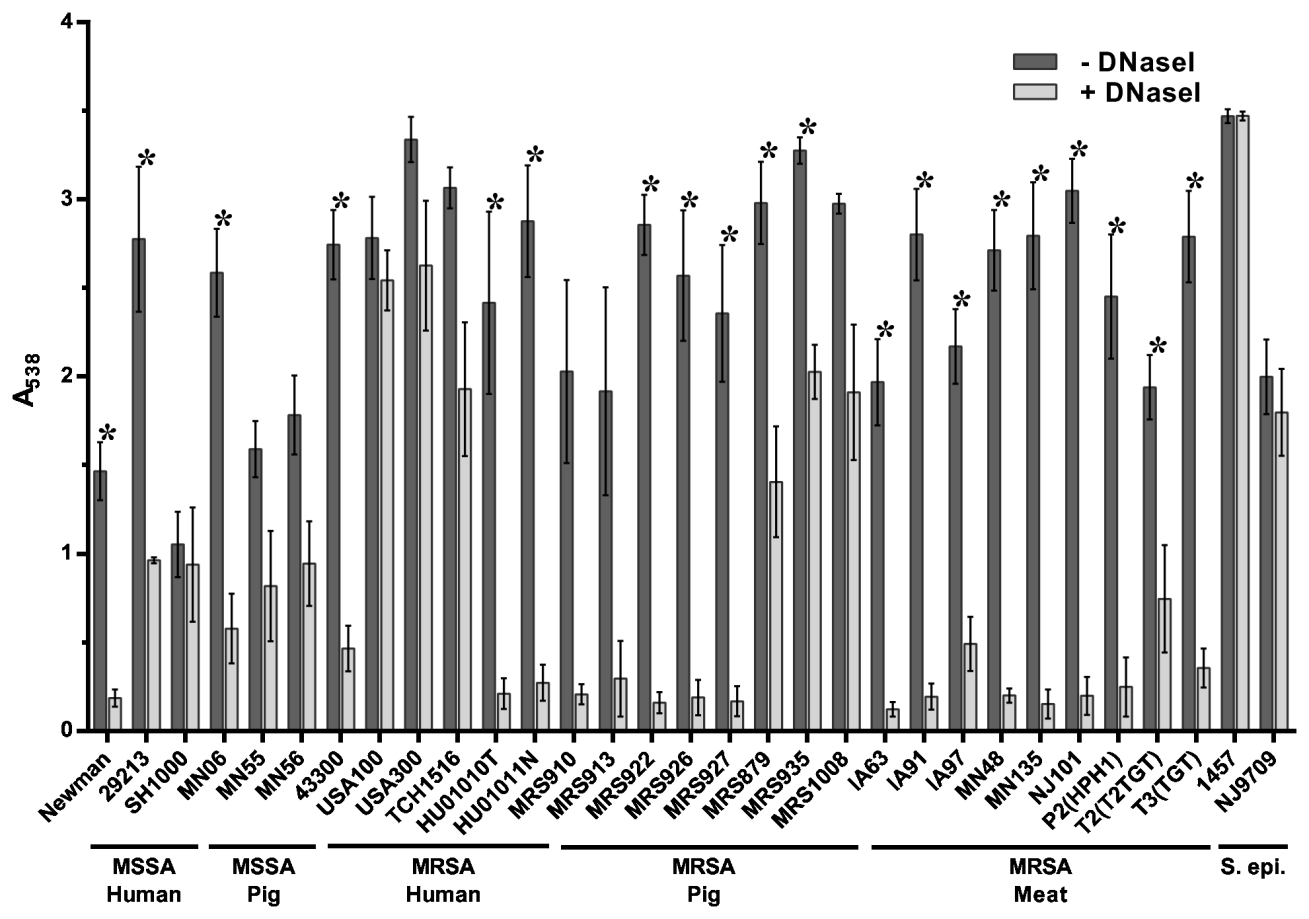
Dispersal of established biofilms by DNaseI. Strains tested are shown along the x-axis and grouped based on methicillin-sensitivity and isolation source. The indicated strains were grown statically for 24 hours to allow biofilm formation. Wells were washed and treated with buffer alone (- DNaseI) or 140 U/ml DNaseI (+ DNaseI) for 2 hours. Biofilm formation was then quantified by standard microtiter assays and measuring the absorbance at 538 nm, plotted along the y-axis. Bars represent the average absorbance obtained from at least 3 independent plates representing biological replicates; error bars represent the SEM. Asterisks (*) denote a *p*-value less than 0.05 between the treated and untreated groups.

Treatment of pre-formed biofilms by DNaseI, shown in Figure 6, had a varying effect on biofilm dispersal. Similar to the inhibition assay, a range of sensitivity to dispersal by DNaseI was observed. As shown in Table 3, biofilm dispersal by DNaseI ranged from near complete (greater than 90% reduction in biofilm biomass) to very little dispersal (USA100, SH1000, USA300, MRS1008, TCH1516 and MRS935 showed a less than 40% reduction in biofilm biomass). The biofilms formed by the ST398 strains were all moderately to highly sensitive to dispersal by DNaseI (Figure 6 and Table 3). In contrast, strains from other STs, including swine isolates (such as MN55, MN56, MRS935 and MRS1008) formed biofilms that were much less sensitive to dispersal by DNaseI (Figure 6 and Table 3). These data suggest that the role of eDNA in the development of late stage biofilms varies between *S. aureus* strains and LA-MRSA STs.

**Figure 7:**
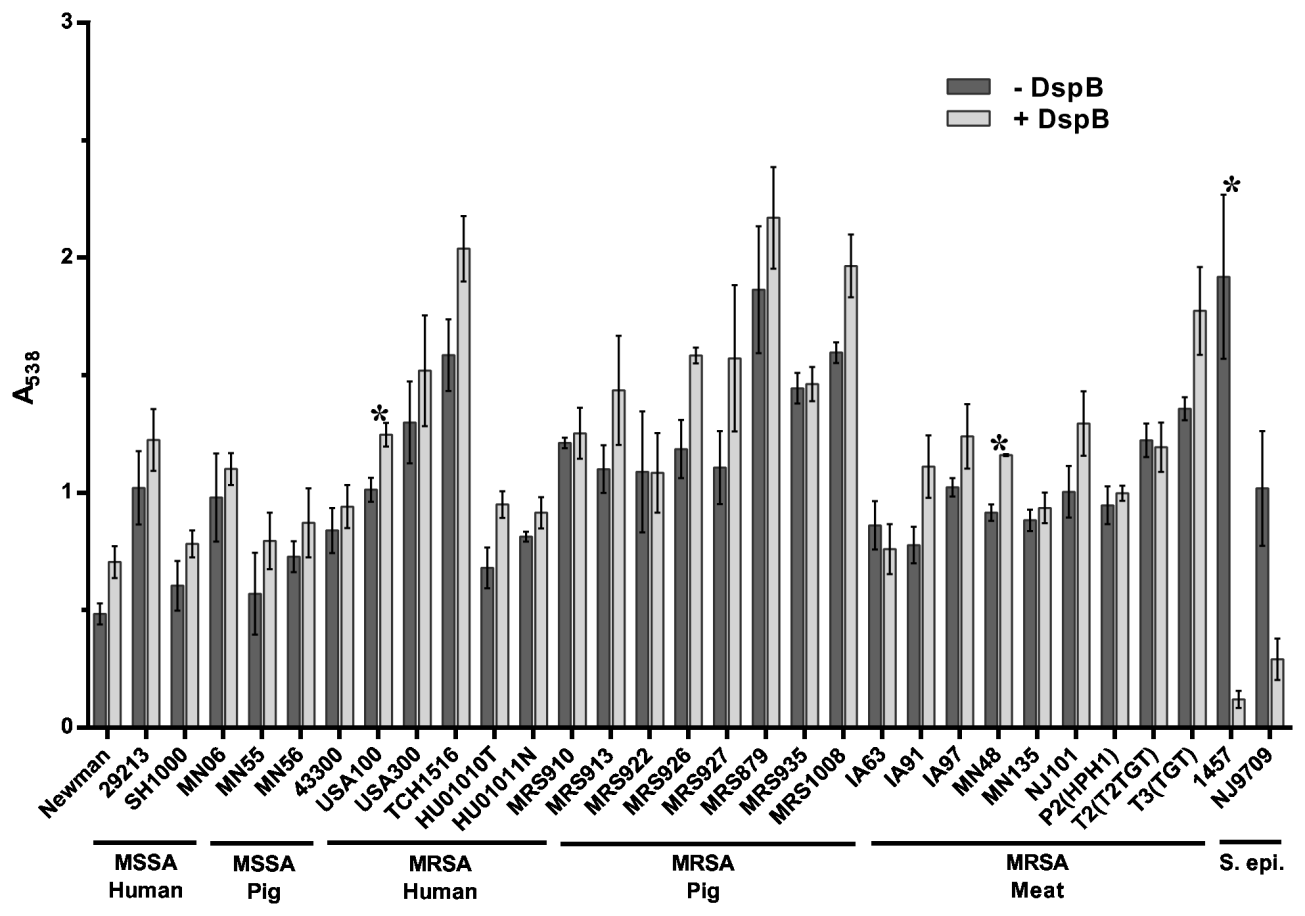
Dispersal of established biofilms by DspB. *S. aureus* strains tested are shown along the x-axis and grouped based on methicillin-sensitivity and isolation source. *S. epidermidis* (S. epi) strains tested are shown along the x-axis and grouped together. Wells were washed and treated with buffer alone (- DspB) or 40 µg/ml DspB (+ DspB) for 2 hours. Biofilm formation was then quantified by standard microtiter assays and measuring the absorbance at 538 nm, plotted along the y-axis. Bars represent the average absorbance obtained from at least 3 independent plates representing biological replicates; error bars represent the SEM. Asterisks (*) denote a *p*-value less than 0.05 between the treated and untreated groups.

Figure 7 shows the results after a 2-hour treatment of pre-formed biofilms with DspB. Treatment with DspB did not disperse the biofilms formed by any of the *S. aureus* strains tested (Figure 7). In contrast, the *S. epidermidis* strains 1457 and NJ9709 formed biofilms that were highly sensitive to this enzyme and showed significant dispersal. The swine isolates showed no difference in sensitivity to DspB from the human isolates or laboratory strains. Consistent with previous findings, these results demonstrate a differential sensitivity to DspB in *S. aureus* compared to *S. epidermidis*.

### Gene expression in biofilms

**Figure 8:**
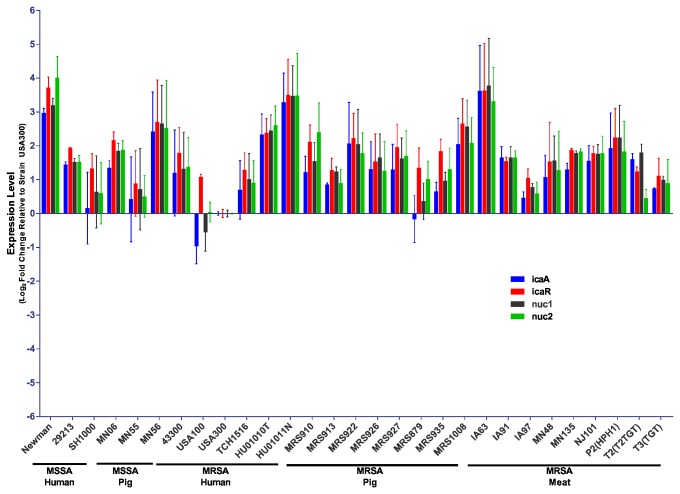
Gene expression. Quantitative real-time PCR was used to determine mRNA expression of *icaA, icaR, nuc1* and *nuc2* in the indicated *S. aureus* strains relative to strain USA300. Each gene was normalized to the expression of the 16S rRNA and fold change is plotted as the mean of two experiments. Error bars represent the SEM.

To further characterize the biofilms formed by the LA-MRSA strains, we performed quantitative real-time PCR (qPCR) using RNA isolated from mature biofilms. Specifically, we were interested in evaluating expression of genes potentially involved in the production of extracellular matrix components. The polysaccharide PNAG is the product of four enzymes encoded by the *icaADBC* operon; expression of this locus is highly regulated by numerous transcription factors, including the negative regulator IcaR [68]. The *icaR* gene is located immediately upstream of *icaADBC*, however it is divergently transcribed [69]. Extracellular nucleases, encoded by the *nuc1* and *nuc2* genes [70], have been proposed to impact the accumulation of extracellular DNA in the biofilm matrix [61,71,72]. Expression of genes involved in PNAG production (*icaA, icaR*) and extracellular nuclease (*nuc1, nuc2*) was measured in biofilms of the *S. aureus* strains and compared to expression in strain USA300 (Figure 8). No statistically significant difference in expression of *icaA, icaR, nuc1*, or *nuc2* was seen across the panel of strains.

### Extracellular protease activity

**Figure 9:**
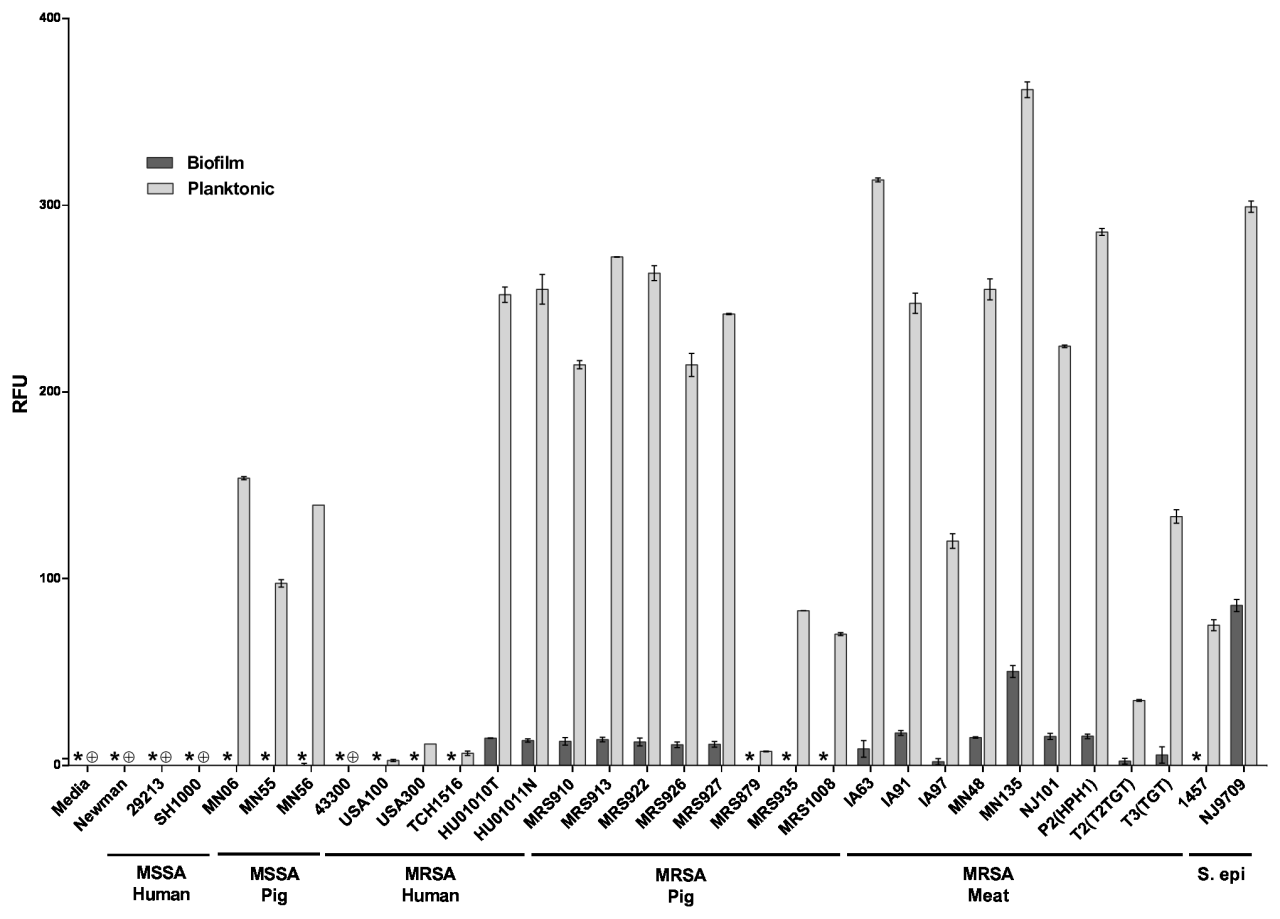
Secreted protease activity. Protease activity present in the culture media was measured using a fluorescent assay. Strains tested are shown along the x-axis and grouped based on methicillin-sensitivity and isolation source. The indicated strains were grown for 22 hours as biofilm or planktonic cultures. Bars represent the average fluorescence obtained from at least 3 independent plates representing biological replicates; error bars represent the SEM. Media: sterile culture medium. *: Not Detected (Biofilm cultures) **⊕**: Not Detected (Planktonic cultures).

There are 10 extracellular proteases produced by *S. aureus*, which have been proposed to act on microbial surface proteins as well as on host proteins [73,74]. We measured extracellular protease activity in the conditioned medium from biofilm and planktonic cultures, to determine if there were any differences between strains. As shown in Figure 9, in the majority of strains, little to no protease activity was detected in the biofilm culture medium. In contrast, for most strains, the planktonic culture medium had measurably higher levels of protease activity. The magnitude of this activity varied across the strains tested, and among the *S. aureus* strains, generally correlated with MLST type: the ST5, ST8 and ST9 strains had lower levels of protease activity than the ST398 strains. In four strains (Newman, 29213, SH1000 and 43300), no protease activity was detected in the conditioned medium from either biofilm or planktonic cultures. *S. aureus* strain MN135 and *S. epidermidis* strain NJ9709 each had a modest level of protease activity in the biofilm culture medium, albeit significantly less than in their respective planktonic culture medium.

### Extracellular nuclease production

**Figure 10:**
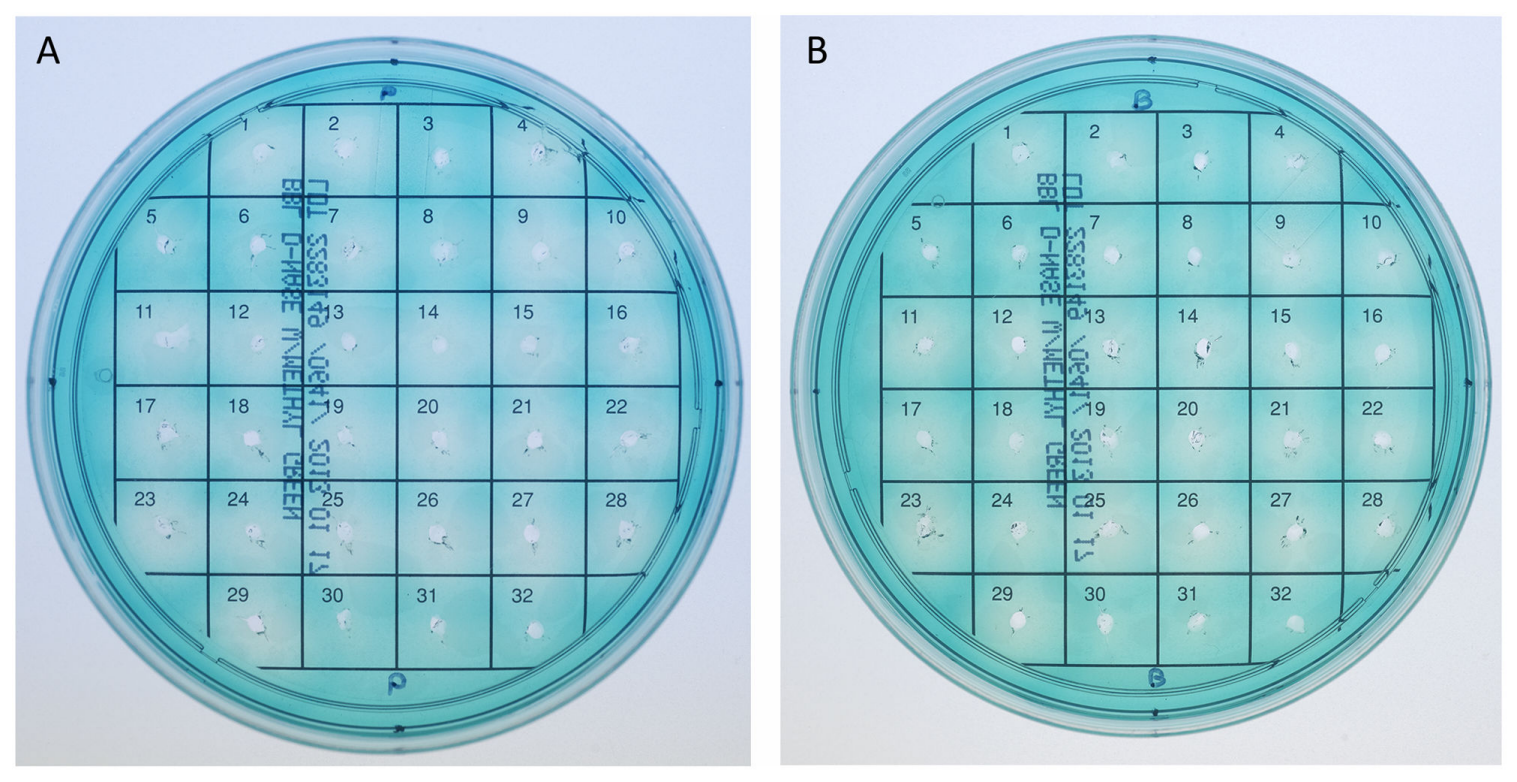
Extracellular nuclease activity. DNase test agar with methyl green was used to detect nuclease activity in planktonic (A) or biofilm (B) cultures. Grid positions: 1. Newman; 2. 29213; 3. SH1000; 4. MN06; 5. MN55; 6. MN56; 7. 43300; 8. USA100; 9. USA300; 10. TCH1516; 11. HU01010T; 12. HU01011N; 13. MRS910; 14. MRS913; 15. MRS922; 16. MRS926; 17. MRS927; 18. MRS879; 19. MRS935; 20. MRS1008; 21. IA63; 22. IA91; 23. IA97; 24. MN48; 25. MN135; 26. NJ101; 27. P2(HPH1); 28. T2(T2TGT); 29. T3(TGT); 30. 1457; 31. NJ9709; 32. Concentrated sterile culture medium.

As extracellular DNA is an important component of *S. aureus* biofilms, we tested the strains for production of secreted nucleases when grown as biofilm and planktonic cultures. A clear zone surrounding the point of inoculation was observed for the majority of the *S. aureus* strains in both biofilm and planktonic culture medium (Figure 10), indicating the presence of a secreted nuclease in the samples. A small number of *S. aureus* strains exhibited low nuclease activity in both culture types: SH1000, MN55, MN56 and USA100. The remaining strains, including all ST398 strains, exhibited higher nuclease activity in both biofilm and planktonic culture medium. The *S. epidermidis* strains 1457 and NJ9709 did not produce a secreted nuclease as expected, since *S. epidermidis* does not possess the *nuc* genes [75]. Comparing conditioned planktonic culture medium (Figure 10A) to conditioned biofilm culture medium (Figure 10B) showed that for all strains that the presence or absence of nuclease activity was the same in both culture types, i.e. if a strain produced nuclease in planktonic culture, it also produced it in biofilm culture. Interestingly, the strains with the least detectable nuclease activity (*S. aureus* SH1000, MN55, MN56, USA100, *S. epidermidis* 1457 and NJ9709), were also among the least sensitive to biofilm formation inhibition and biofilm dispersal by DNaseI (Table 3, Figure 3, Figure 6). 

## Discussion

The spread of MRSA is a serious public health concern for both human and veterinary medicine. LA-MRSA strains, predominately consisting of ST398 isolates, currently represent the largest reservoir of MRSA outside of hospitals [47]. Thus, strategies to eliminate or decrease the prevalence of these strains in swine and other livestock populations are a public health priority. While numerous studies have demonstrated the presence of MRSA ST398 in livestock, there are few studies addressing the virulence properties of these strains. Moreover, to our knowledge, mechanisms contributing to the persistent carriage and high prevalence rates of LA-MRSA strains in swine herds and production facilities have not been investigated. In this report, we tested the ability of swine LA-MSSA and LA-MRSA strains, including ST398, ST9, and ST5, to form biofilms. We then compared the biofilms formed by these strains to biofilms formed by MSSA and MRSA laboratory strains as well as clinical HA-MRSA (USA100) and CA-MRSA (USA300) strains. All LA-MRSA strains tested here formed robust biofilms similarly to human clinical isolates, including two USA300 isolates. Moreover, no statistical differences were observed between any isolates and MLST types tested.

To gain further insight into the mechanisms responsible for biofilm development in LA-MRSA strains, we tested whether enzymes targeting different components of the biofilm matrix (protein, extracellular DNA or the polysaccharide PNAG, respectively) could inhibit biofilm formation, disperse established mature biofilms, or both. Enzymes and enzyme mixtures have been proposed for use in the elimination of biofilms from both abiotic and biotic surfaces; however it is important to take into account the makeup of the particular type of biofilm being targeted [76], as these enzymes can have varying effects on biofilms from different bacterial species and even between strains of a single species [60,77,78]. Additionally, compounds that have been shown to be effective at reducing biofilms of other *Staphylococcus* species, such as *S. epidermidis*, may not be as effective when targeting *S. aureus* biofilms.

Our results demonstrate that Proteinase K inhibited biofilm formation and caused significant detachment of mature biofilms in nearly all *S. aureus* strains tested, including LA-MRSA isolates. Our findings agree with prior results demonstrating the sensitivity of *S. aureus* biofilms to Proteinase K [60,63,76,77,79]. An interesting exception is strain USA300, for which Proteinase K did not inhibit biofilm formation, but was able to disperse mature biofilms. Specifically, we found Proteinase K inhibited biofilm formation in all *S. aureus* strains tested, including TCH1516, a USA300-type strain (ST8, *spa* type t008, community-associated MRSA from humans) isolated from a different source, except for strain USA300, which was the only strain not sensitive to Proteinase K treatment at the time of inoculation. Perhaps this USA300 strain is able to overcome the effect of Proteinase K during biofilm formation by modulating expression of other components during formation of the biofilm matrix. Phenotypic differences such as this can occur even in MRSA strains of the same MLST type and demonstrate that MLST and *spa* type do not indicate a clonal lineage, rather a family of similar strains. The origin of individual MRSA isolates is thought to be the result of multiple evolution events from a progenitor strain and/or divergence and gene acquisition events [80–82]. In contrast to *S. aureus*, it has been shown that biofilm formation and dispersal by a number of *S. epidermidis* strains is not sensitive to Proteinase K or other proteases [76,77]. Similar to these results, we found biofilm formation by *S. epidermidis* strains 1457 and NJ9709 to be insensitive to Proteinase K inhibition and Proteinase K caused little to no detachment in mature biofilms of these strains as well.

Extracellular DNA (eDNA) is another component of the biofilm matrix and the structural role of eDNA in promoting biofilm stability is highly variable and dependent on the bacterial species, growth conditions, and age of the biofilm [61,83–86]. We found DNaseI treatment to have a varying effect on both biofilm inhibition and dispersal. Specifically, when DNaseI was added at the time of inoculation, all of the strains tested displayed a range of sensitivity, from little to no effect to strong, nearly complete inhibition of biofilm formation. DNaseI was observed to have varying effects on the dispersal as well, with some strains showing a much higher degree of sensitivity to this enzyme than others. Both inhibition and dispersal by DNaseI seem to vary among *S. aureus* strains and MLST types indicating that eDNA may be a more significant component in some MLST types of *S. aureus* than in others. The ST398 strains in particular were the most sensitive to both inhibition of biofilm formation and dispersal of pre-formed biofilms by DNaseI, with a greater reduction in biofilm biomass than other non-ST398 strains, including other swine-origin isolates.

The polysaccharide PNAG has been extensively studied as a biofilm matrix component and is a target for the enzyme DspB [52]. PNAG is the product of the *icaADBC* operon, which is highly conserved among *Staphylococcus* isolates [87]. Many studies have shown the importance of this polysaccharide in *S. epidermidis* biofilms, where it is proposed to be the major component of the biofilm matrix, as DspB can inhibit biofilm formation and disperse pre-formed biofilms [59,76,77,88]. However, the role of PNAG in *S. aureus* biofilms is less clear, as studies have shown that some strains of *S. aureus* produce high levels of PNAG, while others produce little to no PNAG [60]. Additionally, some strains have been shown to be sensitive to biofilm dispersal by DspB whereas other *S. aureus* strains are unaffected by this enzyme [59] or the compound sodium metaperiodate, which breaks down PNAG via an oxidation reaction [60,89]. Our results show that DspB has little effect on both biofilm formation and dispersal in the *S. aureus* strains tested here, regardless of host origin, MLST type or methicillin-resistance. In contrast, the *S. epidermidis* strains tested displayed a high sensitivity to DspB.

Using qPCR, we were unable to detect any significant differences across our panel of *S. aureus* strains in the expression of four genes believed to be important for biofilm formation (*icaA, icaR, nuc1*, and *nuc2*). The lack of a difference in *icaA* or *icaR* expression is consistent with our findings that all of the *S. aureus* strains tested responded in a similar manner to treatment with DspB in the inhibition and dispersal assays. Despite observing an association between nuclease production and sensitivity to biofilm inhibition and dispersal by DNaseI, we were unable to identify any significant differences in the expression of *nuc1* or *nuc2* mRNA during biofilm formation.

To address the possibility that the dispersal enzymes (Proteinase K, DNaseI or DspB) added to the cultures during and after biofilm formation may have been degraded by secreted proteases, we measured the level of protease activity present in the conditioned media from biofilm and planktonic cultures. In all strains, protease activity was markedly higher in the planktonic culture medium than in the biofilm culture medium. In the majority of strains, protease activity was barely detectable or undetectable in the biofilm culture medium. The low level of protease activity detected in the biofilm cultures, coupled with the high level of sensitivity to Proteinase K in the *S. aureus* strains is consistent with the conclusion that proteins form a major structural component of the biofilm matrix in these strains. Two strains tested had moderately elevated protease activity in the biofilm culture medium compared to the other strains; it is unclear what the significance of this elevated level is in the *S. aureus* strain MN135, as this strain was sensitive to inhibition and dispersal by Proteinase K. The *S. epidermidis* strain NJ9709 had moderate protease activity in the biofilm culture medium. However, biofilm formation and dispersal by this strain was insensitive to Proteinase K and highly sensitive to DspB, indicating the importance of the polysaccharide component of the matrix as opposed to proteinaceous material. Since the inhibitor enzymes added to the MN135 and NJ9709 biofilm cultures in the inhibition assays were still able to function as biofilm formation inhibitors (Proteinase K, DNaseI with MN135 and DspB with NJ9709), it is unlikely that there was significant proteolytic degradation of the inhibitor enzymes during incubation with the biofilm cultures. This suggests that resistance to these inhibitors on the part of any particular strain was not due to degradation of the inhibitor enzyme.

Detection of extracellular proteases at a much higher level in the planktonic culture medium is consistent with prior reports that expression of extracellular proteases is maximal in stationary phase cultures *in vitro* [90,91]. In *S. aureus*, production of the extracellular proteases is tightly controlled, subject to positive regulation by *agr* and negative regulation by SarA [73]. Numerous reports have shown that biofilm production in *S. aureus* is regulated by these same pathways, with SarA stimulating biofilm formation and *agr* promoting biofilm dispersal [50,57,58,92]. High levels of extracellular protease production have been shown to reduce biofilm formation [73,93], possibly by degrading cell wall-associated proteins such as fibronectin-binding proteins (FnBPA and FnBPB) [79,94]. Our results demonstrating minimal extracellular protease production during growth as a biofilm coupled with higher protease production during planktonic growth are consistent with proposed mechanisms whereby the extracellular proteases participate in the biofilm dispersal process through induction of the *agr* system, promoting dissemination of the bacteria and aiding in the transition from an attached to an invasive phenotype *in vivo* [58,73,78,95].

Interestingly, the magnitude of the protease activity detected in the planktonic culture medium varied across the strains tested, and this variation appears to correlate with MLST type: the ST398 strains tended to have higher levels of protease activity and the ST5 (USA100, MN06, MRS1008, MRS879, MRS935), ST8 (USA300, TCH1516) and ST9 (MN55, MN56) strains had lower activity. Protease activity was undetected in the laboratory strains Newman, 29213, SH1000 and 43300. Variation in extracellular protease activity in different laboratory strains and clinical isolates has been reported previously, ascribed to differences in the levels of expression of global regulators such as *sarA, agr* and *saeRS* [58,96,97].

Investigation of the ability of the various strains to produce functional extracellular nuclease revealed that the majority of strains tested had detectable nuclease activity during both planktonic and biofilm growth. In our assays, there appears to be an association of nuclease production with sensitivity to biofilm inhibition and dispersal by DNaseI, as the strains that demonstrated little or no nuclease activity were also among the least sensitive to DNaseI-mediated inhibition of biofilm formation and dispersal of established biofilms. The ST398 strains, which were the most sensitive to DNaseI inhibition and dispersal, produced higher levels of extracellular nuclease. This data supports the hypothesis that there is a strain-dependent variation of the importance of eDNA as a component of the biofilm matrix. Accumulation of extracellular DNA occurs through controlled cell death, regulated in *S. aureus* by the lysis-promoting *cidABC* operon and the lysis-opposing *lrgAB* operon [98]. Maintaining a balance of this process is critical for biofilm development, as disruption of *cidA* resulted in reduced biofilm adherence, abnormal biofilm structure and reduced accumulation of extracellular DNA in the biofilm matrix [61,62]. A Δ*lrgAB* mutant, on the other hand, displayed enhanced adherence and greater accumulation of eDNA in the biofilm [61]. Extracellular nuclease activity also impacts accumulation of eDNA in *S. aureus* biofilms, as mutations of *nuc1* and/or *nuc2* have been shown to enhance biofilm formation *in vitro*, leading to thicker biofilms with altered biofilm architecture, and overexpression of *nuc* suppressed biofilm formation [61,71,72]. These results demonstrate that proper control of extracellular nuclease activity is important in development of normal biofilm structure. A biofilm is not a homogenous structure; localized microenvironments exist within the biofilm that result in subpopulations of bacterial cells expressing different physiological states [48,99–101]. As the biofilm grows and matures, distinct three-dimensional structural features develop, typically described as towers and channels. Formation of these structures has been linked to controlled cell death and lysis in a number of bacterial species and spatial and temporal regulation of *cid* and *lrg* expression has been demonstrated in *S. aureus* biofilms [55,102,103]. In *S. aureus* biofilms eDNA is predominately associated with the tower structures and mutations in *cidA, lrgAB* or *nuc* altered the distribution of eDNA throughout the biofilm [61,102]. The extracellular nuclease activity detected in our biofilm cultures may function alongside the *cid/lrg* system to modulate the accumulation of eDNA and help maintain proper biofilm structure.

Different laboratories have reported conflicting results concerning the composition of the biofilm matrix and its sensitivity to various enzymatic treatments. In particular, the role of the PNAG polysaccharide has been disputed. Early investigations in *S. aureus* identified the presence of the *ica* locus and production of PNAG as crucial for biofilm formation [69]. Later work demonstrated the presence of proteins and eDNA in the *S. aureus* biofilm matrix [59,77,79,104]. The relative importance of these three factors, polysaccharide, protein and eDNA, has been a matter of some debate and has been shown to vary depending on the specific strains tested and the biofilm growth conditions. In particular, media composition appears to strongly influence the composition of the biofilm matrix [60,79,105]. For these experiments, we chose to focus on a single growth condition, using tryptic soy broth (TSB) supplemented with 0.5% glucose and 3% NaCl as the media and polystyrene plates coated with 20% porcine plasma, as this condition allowed strong biofilm growth for all strains tested and has been used by other investigators. These conditions are believed to replicate the conditions that the organism may encounter *in vivo* [97], particularly the presence of host protein factors on the colonizing surface (supplied by the plasma) and the mildly acidic environment of the host skin [106] (addition of glucose leads to the acidification of the culture medium [79]). Evaluation of other growth conditions was beyond the scope of this investigation. As such, with the exception of increased sensitivity to DNaseI treatment, we found few differences between the LA-MRSA strains and the human MRSA and MSSA strains. In particular, we did not observe a difference in DspB and Proteinase K sensitivity between MRSA and MSSA strains (regardless of origin) as has been reported previously [60]. However, we grew all strains and performed all enzymatic treatments in a single media type, whereas the previous report that showed differential sensitivity to Proteinase K and sodium metaperiodate, which breaks down polysaccharides like PNAG, was performed using different media for growth of MRSA strains and MSSA strains [60].

In conclusion, our data demonstrate that the LA-MRSA strains (ST398 and others) are capable of forming biofilms and that these biofilms have similar characteristics to other *S. aureus* biofilms, including those formed by community-associated and hospital-associated MRSA strains. While this shared phenotype doesn’t contribute to the understanding of other distinguishing features such as host adaption observed among these strains, it does provide a foundation for designing measures to reduce their prevalence. Specifically, approaches used to mitigate biofilms formed by HA-MRSA strains could possibly be applied to mitigate biofilms formed by LA-MRSA strains. Of the LA-MRSA strains tested, we found ST398 strains to be the most sensitive to both inhibition of biofilm formation and dispersal of pre-formed biofilms by DNaseI. Additionally, we found Proteinase K to both inhibit biofilm formation and disperse mature biofilms in all LA-MRSA strains tested. Together, these data serve as a critical first step in designing strategies to eliminate or reduce the spread of MRSA within livestock populations and between livestock and humans.

## Supporting Information

Figure S1 Biofilm formation on plasma coated microtiter plates. Strains tested are shown along the x-axis and grouped based on methicillin-sensitivity and isolation source. The indicated strains were grown statically for 24 hours in tryptic soy broth medium supplemented with 0.5% glucose and 3% NaCl on microtiter plates pre-coated with either 20% human plasma or 20% porcine plasma. Biofilm formation was quantified by standard microtiter plate assay and measuring the absorbance at 538 nm, plotted along the y-axis. Bars represent the average absorbance obtained from at least 3 independent plates representing biological replicates; error bars represent the SEM.
(EPS)
