# Correction: Livestock-Associated Methicillin-Resistant *Staphylococcus aureus* (LA-MRSA) Isolates of Swine Origin Form Robust Biofilms

**DOI:** 10.1371/annotation/6b2dd528-d9e8-41e1-ae58-db15f5936f2e

**Published:** 2013-08-29

**Authors:** Tracy L. Nicholson, Sarah M. Shore, Tara C. Smith, Timothy S. Frana

There was an error in the name of the fourth author.

The correct name is: Timothy S. Frana

The correct citation is: Nicholson TL, Shore SM, Smith TC, Frana TS (2013) Livestock-Associated Methicillin-Resistant *Staphylococcus aureus* (LA-MRSA) Isolates of Swine Origin Form Robust Biofilms. PLoS ONE 8(8): e73376. doi:10.1371/journal.pone.0073376 

